# Ectopic MYBL2-Mediated Regulation of Androglobin Gene Expression

**DOI:** 10.3390/cells13100826

**Published:** 2024-05-11

**Authors:** Antonia Herwig, Carina Osterhof, Anna Keppner, Darko Maric, Teng Wei Koay, Ambre Mbemba-Nsungi, David Hoogewijs

**Affiliations:** Department of Endocrinology, Metabolism and Cardiovascular System, University of Fribourg, 1700 Fribourg, Switzerland; antonia.herwig@unifr.ch (A.H.); carina.osterhof@unifr.ch (C.O.); anna.keppner@unifr.ch (A.K.); darko.maric@unifr.ch (D.M.); teng-wei.koay@bioquant.uni-heidelberg.de (T.W.K.); ambre.mbemba-nsungi@uzh.ch (A.M.-N.)

**Keywords:** oxygen binding, globin, transcription

## Abstract

Androglobin (ADGB) is a highly conserved and recently identified member of the globin superfamily. Although previous studies revealed a link to ciliogenesis and an involvement in murine spermatogenesis, its physiological function remains mostly unknown. Apart from FOXJ1-dependent regulation, the transcriptional landscape of the *ADGB* gene remains unexplored. We, therefore, aimed to obtain further insights into regulatory mechanisms governing ADGB expression. To this end, changes in *ADGB* promoter activity were examined using luciferase reporter gene assays in the presence of a set of more than 475 different exogenous transcription factors. MYBL2 and PITX2 resulted in the most pronounced increase in *ADGB* promoter-dependent luciferase activity. Subsequent truncation strategies of the *ADGB* promoter fragment narrowed down the potential MYBL2 and PITX2 binding sites within the proximal *ADGB* promoter. Furthermore, MYBL2 binding sites on the *ADGB* promoter were further validated via a guide RNA-mediated interference strategy using reporter assays. Chromatin immunoprecipitation (ChIP)-qPCR experiments illustrated enrichment of the endogenous *ADGB* promoter region upon MYBL2 and PITX2 overexpression. Consistently, ectopic MYBL2 expression induced endogenous ADGB mRNA levels. Collectively, our data indicate that ADGB is strongly regulated at the transcriptional level and might have functions beyond ciliogenesis.

## 1. Introduction

Globins, a family of heme-containing proteins, play a fundamental role in oxygen transport and storage, enabling aerobic life forms to efficiently regulate oxygen levels and detoxify reactive oxygen species in various physiological contexts [1,2]. Among these, androglobin, the most recently discovered member of the globin superfamily, has gathered increasing attention for its unique structural and functional properties. It is a multimeric protein, which contains an unusual circularly permuted globin domain with unusual features of a heme-binding mechanism [3,4,5], interrupted by a putative calmodulin-binding motif. Additionally, at its N-terminus, ADGB contains a calpain-like protease domain, possibly having a role in the maturation of sperm [6]. Indeed, we showed that androglobin expression is associated with late stages of spermatogenesis, and Adgb-deficient mice display male infertility, as evidenced by a total lack of functional sperm [6]. Moreover, the defects in head shape and acrosome structure visible in Adgb mutant mice strongly suggest a function of Adgb in the maturation of the acrosome. Two independent studies with human data of male infertility patients with variants in the *ADGB* gene further underscore the functional relevance of our data [7,8].

Originally identified in the testis, androglobin’s presence has since been described for a variety of ciliated cell types, such as airway epithelia and ciliated cells forming the blood–brain barrier [9]. This very distinct expression pattern, as well as additional in silico data mining, led to the hypothesis that ADGB is regulated by FOXJ1, one of the main transcriptional drivers of motile ciliogenesis [10]. FOXJ1-dependent regulation of reporter gene activity driven by ADGB regulatory elements indicated FOXJ1-mediated modulation of ADGB transcription. Consistently, ChIP assays confirmed binding of FOXJ1 to the endogenous *ADGB* gene locus, and ectopic FOXJ1 expression resulted in substantially increased endogenous ADGB mRNA levels, further confirming a FOXJ1-dependent regulation of ADGB. The complexity of *ADGB* gene transcription, as revealed by our initial study, prompted us to further explore its transcriptional regulation. In the current study, we employed various complementary approaches and identified MYBL2 as a novel transcriptional regulator of *ADGB* gene expression.

## 2. Materials and Methods

### 2.1. Expression Plasmid Constructs

A library (ID 23827) with transcription factor cDNAs expressed from a pLenti6.2/V5 backbone was obtained from the DNASU plasmid repository [11]. Several plasmids, including pLenti6.2/V5-DEST-MYBL2 and pLenti6.2/V5-DEST-PITX2 constructs, were confirmed by sanger sequencing (Microsynth AG, Balgach, Switzerland). N-terminally FLAG-tagged PITX2 was subcloned by amplifying the PITX2 coding region using primer pairs with *HindIII* and *KpnI* overhangs (Supplementary Table S2). Amplicons were digested and ligated into pFLAG-CMV-6a vector to produce pFLAG-PITX2. For the transcription factor screening approach, every single transfection experiment included the positive control FOXJ1 for reproducible FOXJ1-mediated induction of *ADGB* promoter-driven luciferase activity. In case FOXJ1 did not result in similar induction of *ADGB* promoter-driven luciferase activity, the whole transfection experiment was disregarded.

### 2.2. Mammalian Cell Culture and DNA Transfection

HEK293T and A375 (ATCC CRL-1619) cells were cultured and maintained in DMEM (Sigma, St. Louis, MO, USA), supplemented with 10% heat-inactivated fetal bovine serum (Sigma) and 1% Pen/Strep Glutamine (PAN Biotech, Aidenbach, Germany). Cells were maintained in a humidified incubator at 37 °C and 5% CO_2_. HEK293T cells were transiently transfected by using the calcium-phosphate method, as described before [12,13]. The medium was changed following an overnight incubation. The A375 cells were transiently transfected with jetOPTIMUS^®^ transfection reagent kit (Polyplus-transfection SA, Illkirch-Graffenstaden, France). Briefly, for 6-well plates, the plasmid DNA was mixed with 100 μL/mL of jetOPTIMUS buffer and 1.25 μL/mL jetOPTIMUS reagent and incubated for 10 min before the transfection mix was added to the cells. A medium change was performed immediately before and a second time 4–8 h after transfection.

### 2.3. Luciferase Reporter Gene Assays

*ADGB* promoter elements used in this study are described in [9], with the exception of AP431_Ca and AP431_Cb, which were newly created during this study. An overview of all elements is given in Supplementary Table S2. A total of 500 ng of pGL3-*ADGB* promoter constructs were co-transfected with 25 ng pRL-SV40 *Renilla* luciferase in 12-well plates. To study the effect of MYBL2 or PITX2 on *ADGB* promoter activity, 750 ng pLenti6.2/V5-DEST-MYBL2 or 500 ng pLenti6.2/V5-DEST-PITX2 were co-transfected with the promoter plasmids and the pRL-SV40 *Renilla*. To normalize the total amount of plasmid DNA, an empty pFLAG-CMV-6a vector was co-transfected with the luciferase constructs. HEK293T cells were lysed 48 h after transfection with 125 μL passive lysis buffer (Promega, Madison, WI, USA), incubated on ice for 15 min, and frozen at −20 °C. The lysates were thawed on ice and pipetted into a 96-well plate (15 μL per sample). Assays were performed according to the Dual-Luciferase^®^ Reporter Assay System kit (Promega), as described before [14,15]. The luminescence was measured (Centro LB960 luminometer Version 5.17, Bad Wildbad, Germany), and the relative firefly/*Renilla* luciferase activities were calculated; luminescence is displayed as relative luminescence units (RLU).

### 2.4. dCas9-Mediated Interference of Transcription Factor Binding

Candidate gRNAs targeting the first 100 bps upstream of the *ADGB* promoter [16], as well as control gRNAs targeting more distal regions, were used to interfere with the binding of MYBL2. For this purpose, 750 ng pLenti6.2/V5-DEST-MYBL2 were co-transfected with 1000 ng pcDNA3-dCas9, 600 ng gRNA, 500 ng of the *ADGB* promoter fragment pGL3-AP431 (and 25 ng of pRL-SV40 *Renilla* into HEK293T cells (12-well plates). Interference of MYBL2 and PITX2 binding was determined by dual luciferase assays.

### 2.5. Chromatin Immunoprecipitation (ChIP)

Chromatin immunoprecipitation was performed as described previously [9,17], with minor modifications. Briefly, 5 × 10^6^ HEK293T cells were seeded in 15 cm dishes and transfected with 10,000 ng pLenti6.2/V5-DEST-MYBL2, pFLAG-PITX2, or pFLAG-CMV-6a plasmid DNA. Moreover, 24 h after transfection, the protein–DNA complexes were cross-linked and fixed for 10 min at room temperature with 1% formaldehyde under gentle agitation. Fixation was stopped by adding 125 mM of glycine in PBS for 10 min. Cells were washed twice, scraped down in PBS, and centrifuged at 1600× *g* at 4 °C for 5 min. Next, the cells were resuspended and lysed in lysis buffer (1% SDS, 2.2 mM EDTA, 165 mM NaCl, and 22 mM Tris-HCL pH 7.5) for 10 min. To generate genomic DNA fragments between 100 and 500 bp, the lysates were sonicated with the Bioruptor Pico (Diagenode, Seraing, Belgium) on the “1.5 mL Tubes frequency” setting for ten cycles (30 s ON/30 s OFF) at 4 °C. The lysates were then centrifuged for 2 min at 16,000× *g* at 4 °C to remove insoluble matter.

For the immunoprecipitation, samples were diluted 1:10 (1.1% Triton X-100, 2 mM EDTA, 20 mM Tris-HCL pH 7.5, 150 mM NaCl) and incubated with either 2 µg rabbit polyclonal anti-IgG as negative control, 2 µg rabbit polyclonal anti-B-Myb (MYBL2) (18896-1-AP, Proteintech Group Inc., Rosemont, IL, USA), 2 µg rabbit polyclonal anti-PITX2 (HPA050074, Sigma, St. Louis, MO, USA), or 2 µg rabbit polyclonal anti-FLAG (20543-1-AP, Proteintech Group Inc.). Immunoprecipitation was performed using the Protein G Mag Sepharose beads (Cytiva GmbH, Freiburg i.Br., Germany). DNA–antibody complexes were washed serially with wash buffers (WB) 1 to 4 (WB1: 0.1% SDS, 1% Triton X-100, 2 mM EDTA, 20 mM Tris-HCl pH 7.5, 150 mM NaCl; WB2: 0.1% SDS, 1% Triton X-100, 2 mM EDTA, 20 mM Tris-HCl pH 7.5, 500 mM NaCl; WB3: 1% Igepal, 1 mM EDTA, 20 mM Tris-HCl pH 7.5, 250 mM LiCl, 0.5% Sodium Deoxycholate; WB4: 20 mM Tris-HCl pH 7.5, 150 mM NaCl, 2 mM EDTA). De-crosslinking of chromatin–protein complexes was performed by overnight incubation at 65 °C and 1500 rpm shaking in elution buffer (20 mM Tris-HCL pH 7.5, 2 mM EDTA, 150 mM NaCl, and 1% SDS). The isolation of the DNA was performed according to the MN NucleoSpin PCR and DNA clean-up Kit (Macherey-Nagel, Hoerdt, France), with the modification that NTB buffer (5 volumes) in combination with isopropanol was used instead of the NTI buffer. To quantify the coprecipitated DNA, a ChIP-qPCR was conducted using primers targeting the *ADGB* promoter, as well as two control regions upstream and downstream of ADGB on chromosome 6 and a positive control targeting the Aurora kinase A (AURKA) promoter region. For this purpose, 3 µL of chromatin was loaded in triplicates. ChIP-seq data described below were used to design primers targeting the peak regions of AURKA after a pulldown with MYBL2. All primers are listed in Supplementary Table S3.

### 2.6. Protein Extraction and Immunoblotting

Cells were washed with PBS and lysed with Triton buffer (50 mM HEPES, 150 mM NaCl, 1 mM EDTA, 10% glycerol 1% Triton X-100, phenylmethylsulphonyl fluoride [PMSF 100 µM], leupeptin, aprotinin, and pepstatin [LAP, 2 μg/mL]). The lysed cells were scratched down, collected into 1.5 mL tubes, and incubated for 15 min on ice, followed by centrifugation for 10 min at 4 °C at 14,000× *g*. The supernatant was collected, and the concentration was measured by Bradford assay in spectrophotometry cuvettes. The absorbance was measured using a UV-1280 spectrophotometer (Shimadzu, Kyoto, Japan) at 595 nm to determine the protein concentrations. A total of 25 µg of proteins were separated on 10% acrylamide and transferred onto a nitrocellulose membrane (Amersham Hybond-ECL, GE Healthcare, Chicago, IL, USA). The membranes were blocked in a 5% dried milk solution in TBS-1% Tween for 1 h at room temperature. Membranes were incubated overnight at 4 °C with primary antibodies against rabbit polyclonal anti-B-Myb (MYBL2) (18896-1-AP, Proteintech Group Inc.), rabbit polyclonal anti-PITX2 (HPA050074, Sigma), rabbit polyclonal anti-FLAG (20543-1-AP, Proteintech Group Inc.), or rabbit anti-ADGB (HPA036340, Sigma) and for 1 h at room temperature with HRP-linked secondary antibody (1:10,000) Amersham ECL Rabbit IgG, (NA934, Amersham, Bukinghampshire, UK). The signal was detected using ECL Prime (Amersham) as described before [18]. Detection and quantification of proteins were performed on a C-DiGit^®^ Western Blot scanner and using ImageStudio program Version 5.2.5 (LI-COR Biosciences, Lincoln, NE, USA).

### 2.7. RNA Extraction and RT-qPCR

A375 and HEK293T cells were lysed on ice 24 h after transfection, and RNA was extracted using the RNeasy Mini Kit (Qiagen, Valencia, CA, USA) according to the manufacturer’s instructions, as previously described [19,20]. Synthesis of cDNA was performed on 1.0–1.5 μg RNA using the PrimeScript cDNA synthesis kit (Takara Bio Inc., Kusatsu, Japan) following the manufacturer’s instructions. A total of 21.5 ng of cDNA was loaded in duplicates, and RT-qPCR was performed using SYBRgreen (KAPA Biosystems, London, UK) on a CFX96 C1000 real-time PCR cycler (Bio-Rad Laboratories, Hercules, CA, USA) [21]. mRNA levels were normalized to β-actin, and results were analysed using the ∆∆CT method. Primer sequences are listed in Supplementary Table S3.

### 2.8. In Silico Analysis of Binding Sites

To predict potential binding sites for MYBL2 factor within AP431 (hg38 chr6:146,598,550–146,599,013), AP431_C (hg38 chr6:146,598,874–146,599,013), and AP431_Cmut3 (hg38 chr6:146,598,914–146,598,973) sequences, the TRANSFAC^®^ MATCH algorithm [22,23] implemented into geneXplain^®^ platform 7.2 (https://genexplain.com/genexplain-platform/, accessed on 4 August 2023) was used. The vertebrate human p0.01 profile was employed. With match process DNA sequences as input, a search was conducted for potential transcription factor (TF) binding sites using a library of position weight matrices (PWMs). It then generates a list of identified potential sites and provides a visual representation of their locations within the input sequence. The search algorithm employed in this process relies on two distinct scoring metrics: the matrix similarity score (MSS) and the core similarity score (CSS). These scoring metrics assess the degree of similarity between the input sequence and the PWM, with values ranging from 0.0 to 1.0, where a score of 1.0 signifies a perfect match. The core of each PWM is defined as the first five consecutive positions that exhibit the highest conservation within the matrix. The corresponding tables filtered based on DNA position and TFs names were exported, as well as the consensus DNA-binding sequences for MYBL2 when they were predicted.

### 2.9. ChIP-Sequencing Analysis

The NCBI SRA database (https://www.ncbi.nlm.nih.gov/sra, accessed on 10 July 2023) and the ENCODE repository (https://www.encodeproject.org/, accessed on 10 July 2023) were searched with “MYBL2” as keyword. If processed data were available, mapping and peak files were visually inspected in the IGV browser [24]. If not, raw data were downloaded and quality-filtered with BBDuk of the BBMap suite (https://sourceforge.net/projects/bbmap/). Reads were mapped against the human or mouse reference genome with Hisat2 [25], and peak calling was performed with the MACS2 software [26]. A full list of the datasets can be found in Supplementary Table S5.

### 2.10. Data Analysis

GraphPad PRISM Software version 10.0.0 was used to analyse the data and generate the figures. Values were analysed using one-way or two-way analysis of variance (ANOVA), followed by Bonferroni multiple comparison post hoc test. Results are presented as mean ± SEM of at least three independent experiments. Differences with *p* ≤ 0.05 were considered statistically significant (* *p* < 0.05, ** *p* < 0.01, *** *p* < 0.001)).

## 3. Results

### 3.1. The ADGB Promoter Is Regulated by Multiple Candidate Transcription Factors

In an earlier publication, we have shown that the first 431 base pairs (bp) of the *ADGB* promoter contain a regulatory binding site for FOXJ1 [9]. In the current study, we aimed to identify additional transcription factors that interact with the *ADGB* promoter sequence. Whereas FOXJ1 was chosen because of co-expression and a putative functional connection, we herein employed a fully unbiased screening approach of a library of transcription factors. We co-transfected a total of 477 out of 1056 DNASU expression plasmids [11] with a luciferase reporter gene driven by the *ADGB* promoter sequence in HEK293T cells and analysed their ability to enhance *ADGB* promoter-dependent reporter activity via dual luciferase assays. An expression plasmid for FOXJ1 was included as a positive control for all assays (Supplementary Table S1).

This screening identified a list of putative candidates (among others, CTCF, GSH-2, KAT14, MYBL2, PITX2, SOX2, SOX10) which increased *ADGB* promoter-driven luciferase activity with a comparable intensity to that of the FOXJ1 positive control (Supplementary Table S1 and Figure S1). These expression vectors were confirmed by Sanger sequencing (Microsynth AG, Switzerland). This top-down approach is consistent with our earlier data, illustrating that the first 431 bp upstream of the *ADGB* transcription start site (TSS) display substantial promoter activity.

**Figure 1:**
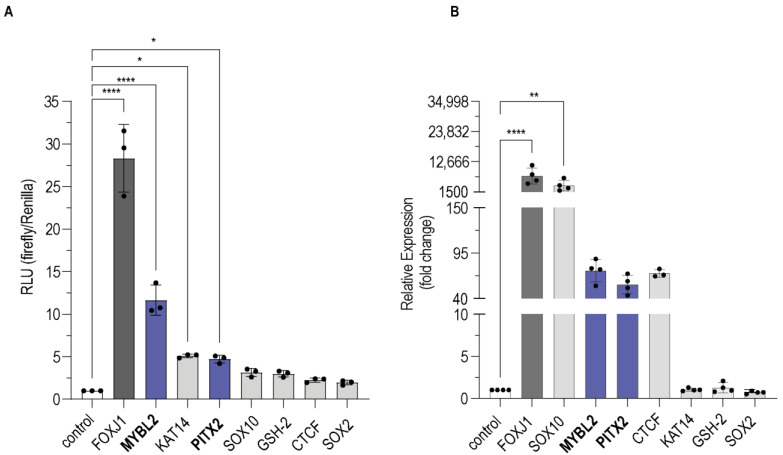
(**A**) Representative overview of transcription factor impact on *ADGB* promoter-driven luciferase activity. Results are displayed in relative luminescence units (RLU) as ratio of firefly to *Renilla* luciferase activities. (**B**) Confirmation of overexpression of transcription factors. We performed qPCR analysis to assess expression intensity of all factors following transfection. RNA samples were DNase-treated before synthesis of cDNA to reduce vector contamination. Results are displayed as relative mRNA expression in fold change. The white bar shows the negative control; black bar shows FOXJ1 as positive control. MYBL2 and PITX2 are highlighted in blue. * *p* < 0.05, ** *p* < 0.01, **** *p* < 0.0001.

Subsequently, we validated these putative factors regarding consistency in the induction of luciferase activity (Figure 1A) and the strength of overexpression (Figure 1B). Whereas KAT14 seemed to induce *ADGB* promoter activity, mRNA analysis did not confirm a convincing overexpression. Similar observations were made for GSH-2 and SOX2. On the other hand, CTCF and SOX10 expression levels were strongly induced upon transient transfection but did not lead to higher levels of *ADGB* promoter activity. After the selection process, only MYBL2 and PITX2 remained as candidates for further validation.

### 3.2. Identification of the Main Binding Region of MYBL2 and PITX2 within the ADGB Promoter

**Figure 2:**
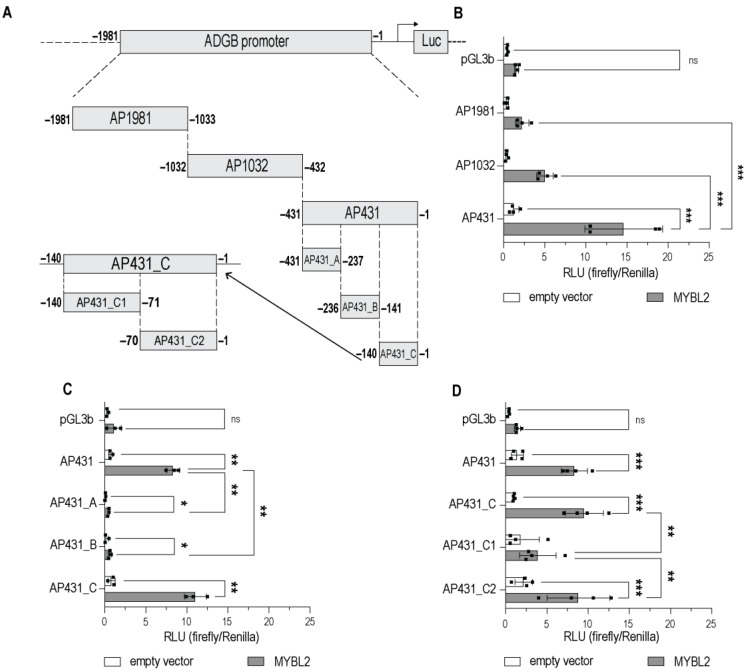
MYBL2 increases promoter activity in the upstream region close to the *ADGB* transcriptional start site (TSS). Luciferase reporter gene assays in HEK293T cells transfected with empty pGL3b vector or various *ADGB* promoter fragments with and without co-overexpression of MYBL2. (**A**) Schematic fragmentation of the *ADGB* promoter. The *ADGB* promoter was systematically fragmented into non-overlapping promoter segments. (**B**) Luciferase reporter gene assay on *ADGB* promoter (AP) fragments in three different lengths: AP431 (−1 bp to −431 bp), AP1032 (−432 bp to −1032 bp), and AP1981 (−1033 bp to −1981 bp) upstream of the *ADGB* TSS. (**C**) Luciferase reporter gene assay on AP sub-fragments of AP431 in three different lengths: AP431_A (−431 bp to −237 bp), AP431_B (−236 bp to −141 bp), and AP431_C (−140 bp to −1 bp). (**D**) Luciferase reporter gene assay on AP sub-fragments of AP431_C: AP431_C1 (−140 bp to −71 bp) and AP431_C2 (−70 bp to −1 bp). Results are displayed in relative luminescence units (RLU) as ratio of firefly to *Renilla* luciferase activities. Data are represented as means ± S.E.M; * *p* < 0.05, ** *p* < 0.01, *** *p* < 0.001, ns = not significant).

As outlined above, the screening (Figure 1) revealed MYBL2 and PITX2, among others, as putative positive regulators of the *ADGB* promoter. We first performed a validation experiment by including two additional *ADGB* promoter fragments (AP1981 and AP1032; Figure 2A), which divided a larger version of the 2 kb *ADGB* promoter into three non-overlapping fragments (Figure 2A). The overexpression of MYBL2 significantly increased *ADGB* promoter-driven luciferase activity for the fragment most proximal to the TSS (AP431), suggesting the binding site of MYBL2 is most likely to be situated in the region between −33 and −431 bp (Figure 2B). Smaller, non-significant inductions could be observed as well in regions further upstream of −431 bp.

The subsequent fragmentation of AP431 into three non-overlapping elements (Figure 2) narrowed down the binding site of MYBL2. The two regions (AP431_A and AP431_B) upstream of −140 bp showed a significant decrease in the MYBL2-dependent induction of *ADGB* promoter-driven luciferase activity compared to AP431, whereas the overexpression of MYBL2 induced the strongest luciferase signal in the *ADGB* promoter fragment most proximal to the TSS, AP431_C (Figure 2C).

AP431_C was further investigated in two different variants (Figure 2D and Supplementary Figure S2A). Variant A represents a division into two non-overlapping fragments (AP431_C1 and AP431_C2; Figure 2D). Using this strategy, we observed a significant MYBL2-dependent induction of fragment AP431_C2 (Figure 2D). Variant B represents a progressive truncation from 140 bp to 60 bp by the stepwise removal of 10 bps from each end (5′ and 3′ end [AP431_C10—AP431_C40, Supplementary Figure S2A variant B]). Whereas this approach did not yield any significant reduction in the MYBL2-mediated activation of *ADGB* promoter activity (Supplementary Figure S2A), further fragmentation of AP431_C resulted in a substantial reduction in the luciferase signal (Supplementary Figure S3A), as both AP431_Ca and AP431_Cb fragments did not display significant MYBL2-dependent increases in luciferase activity.

**Figure 3:**
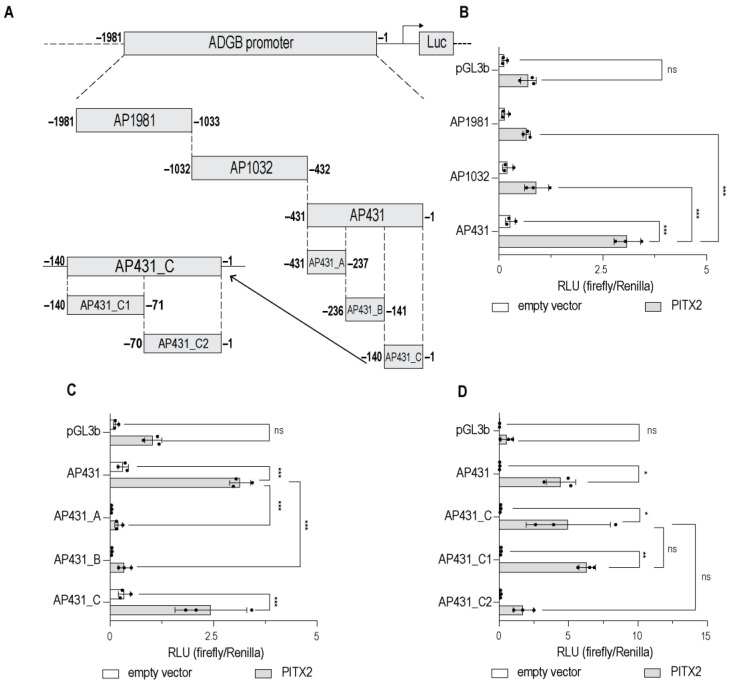
PITX2 increases promoter activity in the upstream region close to the ADGB transcriptional start site (TSS). Luciferase reporter gene assays in HEK293T cells transfected with empty pGL3b vector or various *ADGB* promoter fragments with and without co-overexpression of PITX2. (**A**) Schematic fragmentation of the *ADGB* promoter. The *ADGB* promoter was systematically fragmented into non-overlapping promoter segments. (**B**) Luciferase reporter gene assay on *ADGB* promoter (AP) fragments in three different lengths: AP431 (−1 bp to −431 bp), AP1032 (−432 bp to −1032 bp), and AP1981 (−1033 bp to −1981 bp) upstream of the *ADGB* TSS. (**C**) Luciferase reporter gene assay on AP sub-fragments of AP431 in three different lengths: AP431_A (−431 bp to −237 bp), AP431_B (−236 bp to −141 bp), and AP431_C (−140 bp to −1 bp). (**D**) Luciferase reporter gene assay on AP sub fragments of AP431_C: AP431_C1 (−140 bp to −71 bp) and AP431_C2 (−70 bp to −1 bp). Results are displayed in relative luminescence units (RLU) as ratio of firefly to *Renilla* luciferase activities. Data are represented as means ± S.E.M; * *p* < 0.05, ** *p* < 0.01, *** *p* < 0.001, ns = not significant).

Transient transfection experiments illustrated that PITX2 also binds predominantly to AP431, as fragments AP1981 and AP1032 displayed significantly weaker PITX2-mediated inductions of *ADGB* promoter activity compared to AP431 (Figure 3B). A non-overlapping fragmentation of AP431 showed a significant PITX2-mediated increase in the more proximal fragment (AP431_C) of the *ADGB* promoter (Figure 3C). Further refinement via the fragmentation of AP431_C (Figure 3A,D) displayed a significant PITX2-mediated increase in the more distal fragment of the *ADGB* promoter (AP431_C1), indicating that the putative PITX2 binding site is located between −140 and −71 bp (Figure 3D). Further fragmentation did not result in a significant reduction in the PITX2-mediated activation of *ADGB* promoter activity (Supplementary Figures S2B and S3C).

Although substantial variability persists, our data collectively indicate a consistent regulation of *ADGB* promoter-driven luciferase activity by MYBL2 and PITX2 and implicates that the core region of the main putative binding sites is located within the first 140 bp of the *ADGB* promoter region (AP431_C).

### 3.3. MYBL2 Binding to the ADGB Promoter Is Impaired by dCas9-Mediated Blocking and Mutation of the Putative Binding Site

**Figure 4:**
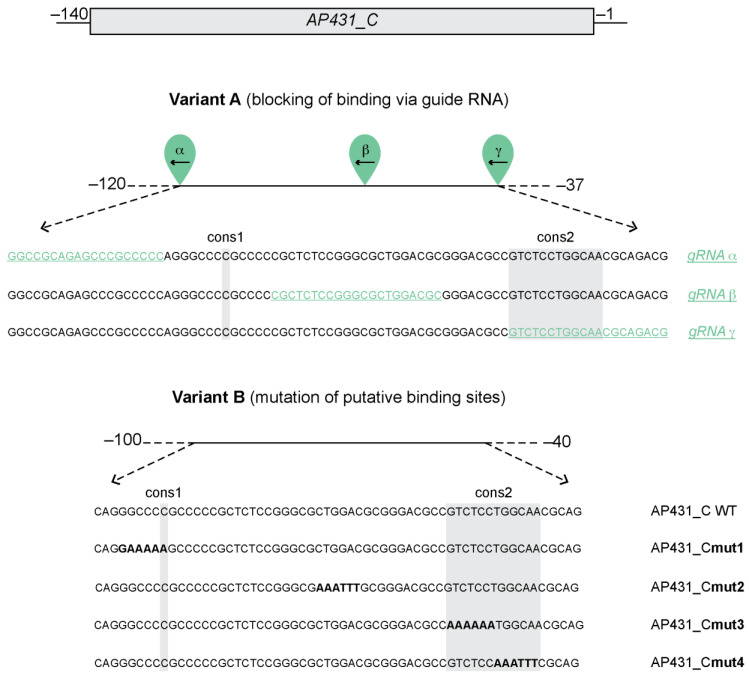
Schematic overview on dCas9-mediated interference strategy and mutation of AP431_C. Schematic overview of both experimental strategies (Variant A and B) to investigate potential MYBL2 binding sites on AP431_C. Variant A, CRISPR/dCas9 gRNAs (α, β, and γ) targeting specific regions on AP431_C (between −120 bp and −37 bp) were used to abolish MYBL2 binding to this region upstream of the *ADGB* TSS. gRNA α (−120 bp to −100 bp), gRNA β (−86 bp to −66 bp), and gRNA γ (−57 bp to −37 bp) are displayed in green and underlined. Variant B shows the locations of the used mutations on AP431_C (−100 bp to −40 bp upstream of the *ADGB* TSS). Substitution-based mutation at −96 to −92 bp (mut1), at −73 to −68 bp (mut2), −57 to −52 bp (mut3), and −51 to −46 bp (mut4), are highlighted in bold. For both variants, two evolutionarily conserved regions (Cons1 and Cons2) are highlighted with a grey background.

**Figure 5:**
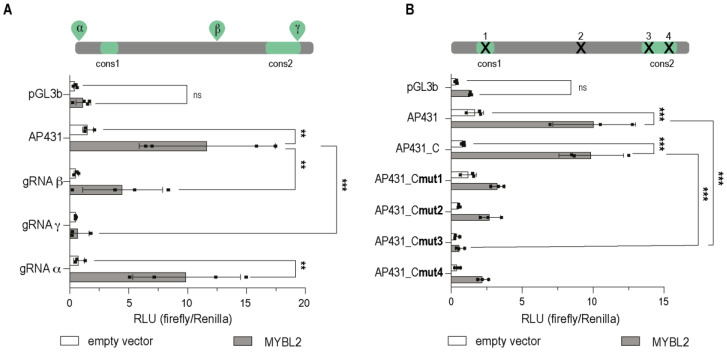
MYBL2-binding to the *ADGB* promoter is impaired by dCas9-mediated interference or mutation of the binding site. (**A**) Luciferase reporter gene assays in HEK293T cells transfected with empty pGL3b vector or AP431 with and without co-transfection of dCas9 and gRNAs (α, β, and γ) and with and without co-overexpression of MYBL2. The gRNA binding sites and conserved sequences are schematically depicted on top. (**B**) Luciferase reporter gene assays in HEK293T cells transfected with empty pGL3b vector or mutated AP431_C with and without co-overexpression of MYBL2. The position of the different mutations and the conserved sites are schematically depicted on top. Results are displayed in relative luminescence units (RLU) as ratio of firefly to *Renilla* luciferase activities. All statistically significant comparisons are shown. Data are represented as means ± S.E.M; ** *p* < 0.01, *** *p* < 0.001, ns = not significant).

To further narrow down the binding site of MYBL2 in the *ADGB* promoter, we employed a CRISPR/dCas9-based approach using a guide RNA (gRNA)-mediated interference strategy (Figure 4). For this purpose, HEK293T cells were transiently co-transfected with a library of *ADGB* promoter-specific gRNAs [9], dCas9, SV40-*Renilla*, *ADGB* promoter-driven reporter gene (AP431), and MYBL2 followed by dual luciferase assays to measure reporter activity. Whereas the co-transfection of gRNA α maintained a MYBL2-dependent increase in *ADGB* promoter activity, gRNA β substantially reduced, and gRNA γ entirely suppressed the MYBL2-mediated increase in promoter activity (Figure 5A). These results suggest that the putative binding site of MYBL2 on the *ADGB* promoter resides within the 20 bp covered and blocked by gRNA γ. To further suggest specificity of gRNA γ, we performed a control without gRNAs, displaying a stable MYBL2-dependent induction of *ADGB* promoter activity (Supplementary Figure S4).

In silico prediction with the TRANSFAC^®^ MATCH algorithm confirmed a putative MYBL2 binding motif in the AP431 fragment (Supplementary Figure S5) that encompasses the binding site of gRNA γ. No binding sites of CDE/CHR, a possible MYBL2 binding element [27,28], could be detected. We hypothesized that the mutation of these predicted MYBL2 binding sites results in a loss of activation capacity, and therefore, we screened all our available mutated fragments. TRANSFAC^®^ showed that all predicted binding sites for MYBL2 on AP431 were eliminated in AP431_Cmut3 (Supplementary Figure S5). Additionally, the evolutionarily conserved regions, referred to as Cons1 and Cons2 (Figure 4), were explored as potential binding sites for MYBL2 within the *ADGB* promoter. Cons1 represents a region on the *ADGB* promoter (−92 bp upstream of the TSS) where a single nucleotide is conserved across several vertebrate species. Cons2 (−57 to −45 bp upstream of the TSS), in contrast, represents a 12 nucleotide-long evidence of evolutional constraint region, as demonstrated before [9] by analysis of PhyloP and PhastCons scores (Figure 4 variant B). To a similar extent as the FOXJ1-dependent activation of the *ADGB* promoter suppressed by the mutation of this region [9], MYBL2-mediated activation is reduced upon the mutation of Cons2. AP431_Cmut3 resulted in the most pronounced suppression of the MYBL2-dependent activation of *ADGB* promoter-driven luciferase activity (Figure 5B), consistent with our results obtained above with gRNA γ.

Collectively, the gRNA-mediated interference strategy and the mutational analyses of the *ADGB* promoter provide insights towards potential MYBL2 binding sites on the *ADGB* promoter. Moreover, it shows that the evolutionarily conserved region Cons2 has a crucial involvement in the binding of MYBL2 on the *ADGB* promoter. The consistency of mutational and gRNA-mediated effects reinforces the conclusion that conserved regions in the *ADGB* promoter play an essential role in regulating ADGB transcription.

### 3.4. MYBL2 and PITX2 Interact with the Endogenous ADGB Promoter

**Figure 6:**
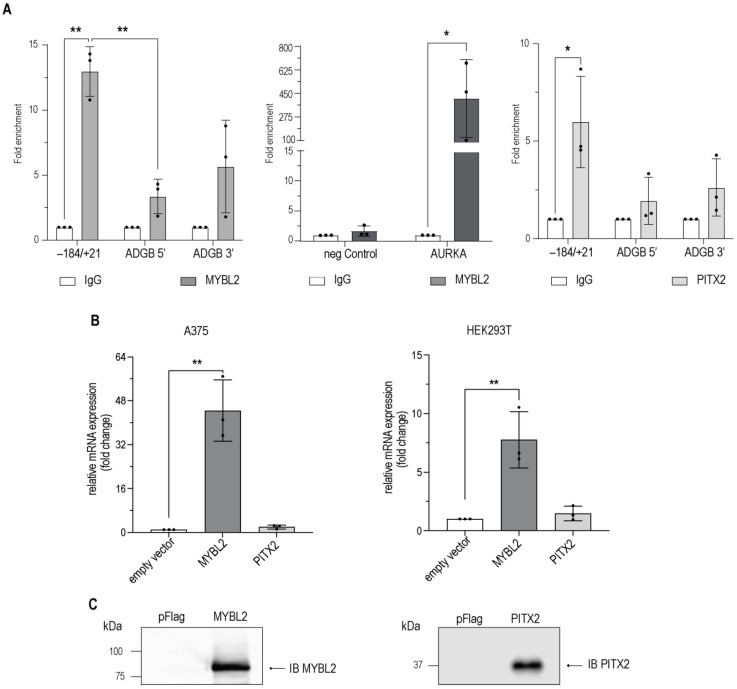
MYBL2 (**left** panel) and PITX2 (**right** panel) interact with the endogenous *ADGB* promoter via direct binding. (**A**) Chromatin immunoprecipitation (ChIP) experiments in HEK293T cells transiently transfected with MYBL2 or FLAG-PITX2. Coprecipitated chromatin derived from the *ADGB* promoter was determined by qPCR using a primer pair covering +21 bp to −184 bp upstream of the *ADGB* TSS. Control regions were targeted upstream (5′ end, ADGB 5′) and downstream (3′ end, ADGB 3′) of ADGB on chromosome 6 and two independent *AURKA* loci (as positive and negative control). Due to the high differences in intensity, statistics for both loci were conducted separately. (**B**) Relative mRNA expression of endogenous ADGB following transient overexpression of MYBL2 or PITX2 compared to empty vector in A375 cells or HEK293T cells. (**C**) Overexpression of MYBL2 (**left** panel) and PITX2 (**right** panel) was verified by immunoblotting using specific antibodies against MYBL2 and PITX2. Data are represented as means ± S.E.M; * *p* < 0.05, ** *p* < 0.01).

To characterize whether MYBL2 interacts with the endogenous *ADGB* promoter region, we first screened publicly available chromatin immunoprecipitation (ChIP) sequencing data. We could detect a weak but statistically non-significant enrichment of *ADGB* promoter DNA in ENCODE datasets from HepG2 cells that encompass the experimentally identified MYBL2 binding site, but we found no signal in K562, indicating a putative cell line-specific binding (Supplementary Figure S6). No signal could be found in the other datasets screened (full list: Supplementary Table S5). To provide more evidence for MYBL2 binding to the *ADGB* locus, we performed ChIP assays in HEK293T cells transiently overexpressing MYBL2. To confirm the functionality of the overexpression system, the same samples were employed to explore potential MYBL2 enrichment at the promoter region of *AURKA*, an established MYBL2 target gene [29,30]. To design a primer pair for this control experiment, we used the enriched region from publicly available MYBL2 ChIP sequencing data. A quantitative PCR analysis displayed profound MYBL2 enrichment at the *AURKA* locus, validating the overexpression system (Figure 6A). In line with the HepG2 ChIP sequencing data, a quantitative ChIP analysis revealed more than tenfold MYBL2 enrichment at the endogenous *ADGB* promoter compared to the IgG antibody control (Figure 6A). Although not as pronounced as the enrichment of the *AURKA* promoter, enrichment at the *ADGB* promoter was highly reproducible. Furthermore, no MYBL2 enrichment was detected in control regions 13 kb upstream and 10.5 kb downstream of the *ADGB* locus on chromosome 6, as well as at an independent *AURKA* locus.

An interaction between PITX2 and the endogenous *ADGB* promoter region was also detected. Quantitative ChIP analysis displayed a sixfold enrichment of PITX2 in the *ADGB* promoter region (Figure 6A). Furthermore, no enrichment was observed in control experiments with empty pFLAG vector-overexpressing HEK293T cells (Supplementary Figure S7). These observations emphasize the targeted binding of MYBL2 and PITX2 to the endogenous *ADGB* promoter and confirm their direct role in the transcriptional regulation of ADGB.

Finally, we explored whether the transient overexpression of MYBL2 in HEK293T and A375 cells, two cell lines which do not express ADGB endogenously [31], regulates endogenous ADGB mRNA levels. RT-qPCR experiments revealed a tenfold increase in ADGB mRNA levels in HEK293T cells (Figure 6B) and an even more pronounced 40-fold increase in ADGB mRNA levels in A375 cells (Figure 6B), providing additional confirmation for a MYBL2-dependent regulation of ADGB expression. In contrast to MYBL2, PITX2 overexpression resulted in a weak, statistically non-significant induction of endogenous ADGB mRNA levels in both cell lines (Figure 6B). Immunoblotting experiments with MYBL2 and PITX2 antibodies confirmed the overexpression of both proteins (Figure 6C). Taken together, these findings suggest that ADGB is regulated by both MYBL2 and PITX2 via direct binding to the endogenous promoter locus.

## 4. Discussion

Androglobin is a highly conserved member of the globin family that is predominantly expressed in ciliated and flagellated cells. This very distinct expression pattern is orchestrated by FOXJ1, an important transcription factor for the differentiation of multi-ciliated cells. In this work, we provide evidence that in addition to FOXJ1, MYBL2 and PITX2 also robustly bind to the endogenous *ADGB* promoter. Extensive efforts were performed to precisely localize the binding sites of MYBL2 and PITX2 on the *ADGB* promoter (Figure 2 and Figure 3). Interestingly, the nucleotides which are crucial for the binding of MYBL2 represent the same evolutionarily conserved sites that FOXJ1 binds to [9]. As MYBL2 binds to an evolutionarily conserved region (−60 to −37 bp upstream of the *ADGB* TSS), our findings considerably expand the existing knowledge of *ADGB* gene regulation.

Additionally, our results suggest a diverse regulatory landscape for the *ADGB* gene locus. MYBL2 alone is sufficient to substantially induce ADGB mRNA expression in A375 and HEK293T, representing a mode of direct regulation. PITX2, however, behaves differently: although we could show robust binding of PITX2 to the *ADGB* promoter via ChIP experiments as well as induction of the *ADGB* promoter via luciferase assays, the overexpression of PITX2 only marginally induces the endogenous expression of ADGB mRNA, possibly indicating a more complex second layer of transcriptional regulation with co-factors that still need to be identified. The presence of histone marks indicative of accessible chromatin within the *ADGB* promoter [9], as well as the lack of modulated ADGB expression levels upon DNA methyltransferase inhibitor 5′-Azacytidine treatment (data not shown), further supports this hypothesis.

By deciphering the regulatory mechanisms that underlie *ADGB* gene expression, this study provides the basis for exploring a broader functional context. The regulation of the *ADGB* gene locus via PITX2 and MYBL2 could indicate that ADGB is involved in processes beyond spermatogenesis [6]. Pitx2 deficiency during early embryogenesis, for example, leads to congenital heart defects and *situs inversus* [32], phenotypes which also occur in ciliopathies derived from both motile and immotile cilia [33]. Adgb mRNA is expressed in the left–right organizer [34], in line with our findings in other cell types carrying motile cilia [9]. A truncated version of FOXJ1 that is no longer able to transactivate the *ADGB* promoter has also recently been shown to cause congenital heart defects [35]. It is, therefore, conceivable that the interaction between PITX2 and ADGB, two factors with otherwise no apparent co-expression (https://www.proteinatlas.org/ENSG00000164093-PITX2/tissue) [31] (Supplemental Figure S8), is temporally restricted to early stages of embryogenesis during the determination of left–right symmetry. Recent work demonstrated that another globin protein family member, cytoglobin, regulates left–right symmetry in zebrafish [36]. Future studies in our Adgb knockout mouse model [6] will show whether Adgb may also play a role in the establishment of left–right symmetry.

Intriguingly, MYBL2 does not seem to be involved in the regulation of ciliogenesis. On the contrary, the activation of MYBL2 promotes cell cycle progression [37,38,39], whereas ciliogenesis mostly occurs when cells are in a quiescent state [40]. Additionally, a well-established downstream target of MYBL2 [30], Aurora kinase A, actively induces the resorption of the primary cilium, thus marking the end of the inert phase [37,38,39,41]. Of note, it has been shown that MYBL2-dependent regulation does not necessarily include direct binding of the transcription factor to its target but rather involves cofactors that bind first and recruit it [42]. We cannot exclude that the MYBL2-dependent regulation we described here is of a similar, indirect nature. Further investigation is needed to determine any cofactors that initiate the MYBL2-dependent activation of the *ADGB* gene.

MYBL2 is expressed in a wide array of tissues and cell types, with the highest values in bone marrow and the testis [41]. The testis are a common expression site of ADGB and MYBL2; however, there is no temporal overlap. Whereas MYBL2 expression is abundant during the early stages of spermatogenesis, ADGB expression peaks much later [6]. It might be possible that slow turnover rates of the transcription factor lead to interactions that are not evident from transcriptional data alone, but the interaction of MYBL2 with the *ADGB* gene locus is probably more relevant in a completely different context. The genetic knockout of Mybl2 is already lethal during gestation [43], which complicates functional analysis beyond this point, but work in a conditional knockout model showed a vital role for Mybl2 in the development of the myeloid lineage [41,44]. In the public databases of the human protein atlas, evidence exists for an occurrence of ADGB mRNA in myeloid dendritic cells derived from this lineage (https://www.proteinatlas.org/ENSG00000118492-ADGB/immune+cell; [45]). The MYBL2-dependent regulation of ADGB, thus, hints at a completely new and ciliogenesis-independent site of expression that requires more investigation.

In conclusion, this study substantially improves the understanding of ADGB transcriptional regulation, further pointing to its complex transcriptional control and potential functional contexts beyond its established role in sperm maturation. Our data highlight the significance of evolutionarily conserved regions in the promoter that are critical for binding and transcriptional activation by regulatory factors. Elucidating the mechanism by which MYBL2 and PITX2 coordinate their actions in vivo, especially in the context of cell-specific expression and the potential interplay with other transcription factors, will be crucial. Since both transcription factors, as well as FOXJ1, bind to the same motif, it is possible that transcription is regulated via the competition of these factors. It will be interesting to study these interactions in suitable cell systems with the modulable endogenous expression of these factors. Additionally, our study raises intriguing questions for future functional investigations. Understanding the consequences of ADGB expression during the establishment of left–right symmetry as well as in immune cells represents major challenges for the future and will require extensive experimental efforts.

## 5. Limitations of the Study

In this study, we provide evidence of the MYBL2- and possible PITX2-dependent regulation of the *ADGB* gene locus. We could show, via several independent methods, that both factors, upon overexpression, bind consistently to both the overexpressed luciferase construct as well as to the endogenous *ADGB* promoter. These findings have new implications for the study of ADGB: MYBL2 expression occurs independent of ciliogenesis and is important for the proper development of the myeloid lineage, which itself could represent an unexpected novel expression site for ADGB.

When interpreting the results of the current study, the following limitations need to be considered. Supplementary Figure S1 and Table S1 contain additional data from our screening analysis. We would like to emphasize the exploratory nature of these experiments and advise caution in interpreting the results for the other unverified targets due to their high variability, which is probably due to experiments that were still subject to optimization. Secondly, while endogenous MYBL2 can be detected (Supplementary Figure S9), HEK293T cells express low to no endogenous levels of PITX2 and may, as such, miss key cofactors that would allow the binding of overexpressed PITX2 to the endogenous *ADGB* promoter, possibly explaining the low PITX2-dependent activation of endogenous ADGB mRNA levels. Thirdly, the overexpression of MYBL2 has been shown to be implicated in altering chromatin accessibility and affecting the binding of pioneer transcription factors, which could alter the cellular phenotype [46]. Fourthly, our study employs the pGL3-based reporter system. Although we could not detect any off-target activity in our validation analysis, the backbone may carry cryptic transcription factor binding sites and might be susceptible to nonspecific transcriptional regulation [47]. Follow-up studies remain to be conducted to further validate that endogenous MYBL2 and PITX2 regulate ADGB expression levels and bind to the *ADGB* promoter-proximal region in cells expressing ADGB endogenously.

## Data Availability

Publicly available datasets were analysed in this study. A list of datasets, as well as accession numbers, can be found in Supplementary Table S5. All other data are contained within the article or Supplementary Materials. Additional statistical analyses, including non-significant comparisons, are available upon request.

## Supplementary Materials

The following supporting information can be downloaded at: https://www.mdpi.com/article/10.3390/cells13100826/s1, Figure S1: Representative overview of transcription factor impact on *ADGB* promoter-driven luciferase activity; Figure S2: MYBL2 and PITX2 increase promoter activity in the upstream region close to the *ADGB* transcriptional start site (TSS); Figure S3: MYBL2 does not increase *ADGB* promoter activity upon fragmentation of the distal upstream region within 140 bp of the *ADGB* TSS; Figure S4: Lack of modulation of *ADGB* promoter-driven luciferase activity by dCas9 in absence of transiently transfected gRNAs; Figure S5: Predicted MYBL2 binding sites in the *ADGB* promoter are lost after mutating AP140 sequence; Figure S6: Analysis of publicly available ENCODE ChIP-sequencing data; Figure S7: Chromatin immunoprecipitation in non-transfected cells displays absence of MYBL2 or PITX2 binding at the *ADGB* locus; Figure S8: Lack of co-expression of ADGB and MYBL2; Figure S9: Endogenous MYBL2 expression in HEK293T; Table S1: screening results; Table S2: primer list cloning; Table S3: primer list RT- and ChIP qPCR; Table S4: mutations and gRNAs; Table S5: ChIP Seq datasets; Table S6: Antibodies. Reference [9] is cited in Supplementary Materials.
