# Supplementary material for: Ectopic MYBL2-Mediated Regulation of Androglobin Gene Expression
Source: Cells. 2024 May 11;13(10):826. doi: 10.3390/cells13100826 (PMC11119863; doi:10.3390/cells13100826)
Supplement: Supplementary file 1 [file cells-13-00826-s001.zip › Supplemental Figures and legends_revised3.pdf]

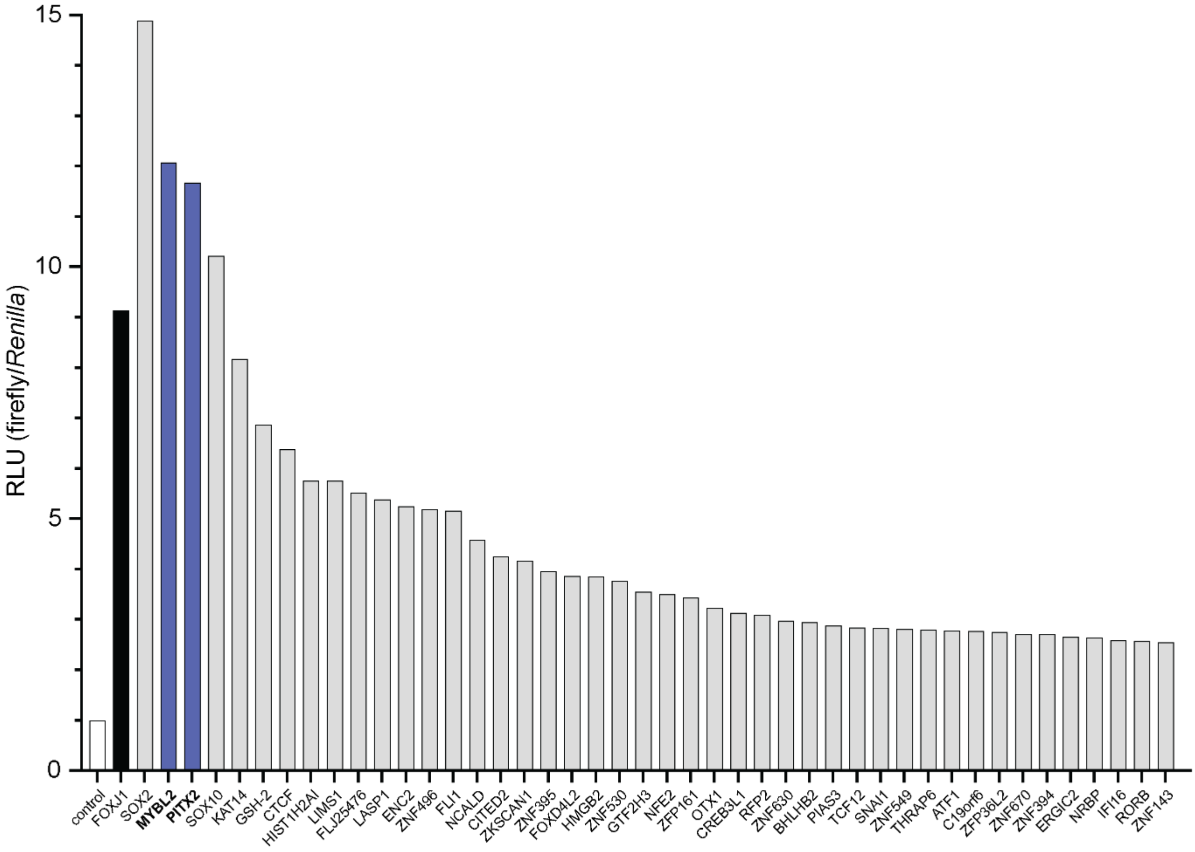

Supplementary figure S1: **Representative overview of transcription factor impact on *ADGB* promoter-driven luciferase activity**. The screening included a total of 477 TFs. The white bar shows the negative control, black bar shows FOXJ1 as positive control. MYBL2 and PITX2 are highlighted in blue as they are chosen for further analysis, following validation in independent assays.

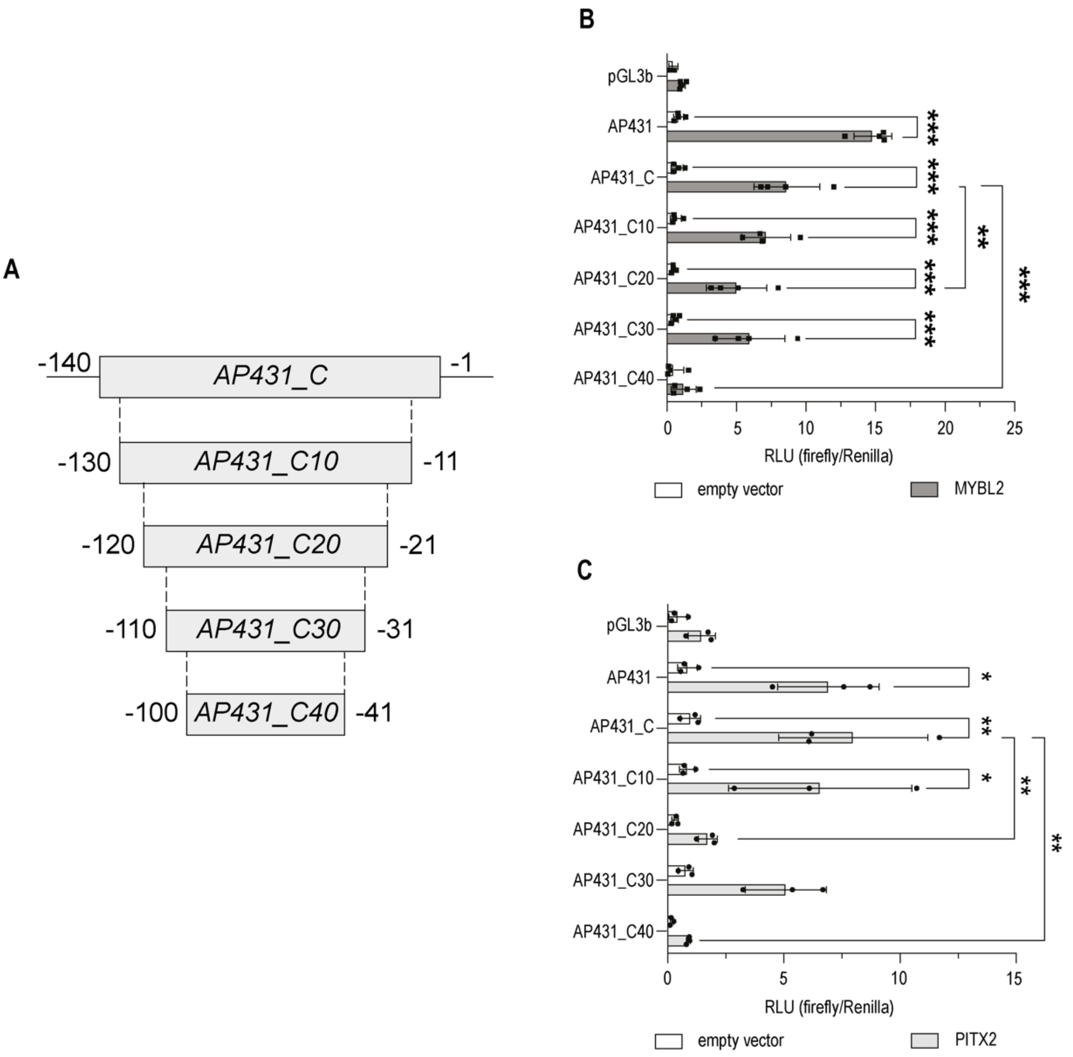

Supplementary figure S2: **MYBL2 and PITX2 increase promoter activity in the upstream region close to the *ADGB* transcriptional start site (TSS).** Luciferase reporter gene assays in Hek293T cells transfected with empty pGL3b vector or various *ADGB* promoter fragments with and without co-overexpression of MYBL2 or PITX2. (A) Schematic fragmentation of the *ADGB* promoter. The *ADGB* promoter fragment AP431_C was progressively truncated (10 bps at each end) into four fragments. Luciferase reporter gene assay on AP sub fragments of AP431_C: AP431_C10 (-130 bp to -11 bp), AP431_C20 (-120 bp to -21 bp), AP431_C30 (-110 bp to -31 bp) and AP431_C40 (-100 bp to -41 bp) with and without co-overexpression of (B) MYBL2 or (C) PITX2. Results are displayed in relative luminescence units (RLU) as ratio of firefly to *Renilla* luciferase activities. All statistically significant comparisons are shown. Data are represented as means ± S.E.M; *$p < 0.05$, **$p < 0.01$, ***$p < 0.001$).

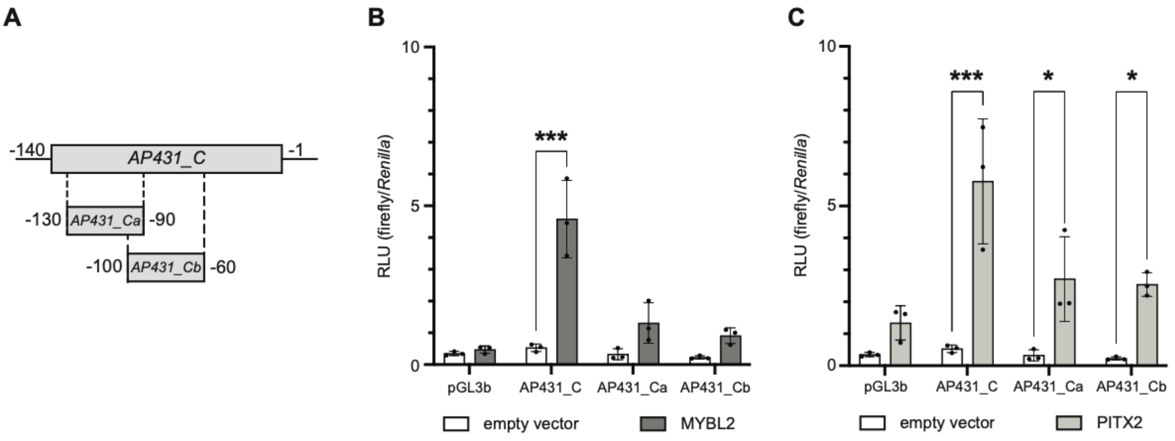

Supplementary figure S3: **MYBL2 does not increase *ADGB* promoter activity upon fragmentation of the distal upstream region within 140 bp of the ADGB TSS. (A)** The AP431_C fragment was sub-fragmented into two further fragments AP431_Ca (-130 bp to -90 bp upstream of the *ADGB* TSS) and 431_Cb (-100 bp to -60 bp upstream of the *ADGB* TSS). Luciferase reporter gene assays in Hek293T cells transfected with empty pGL3b vector, AP431_C, AP431_Ca or AP431_Cb with and without co-overexpression of **(B)** MYBL2 or **(C)** PITX2. All statistically significant comparisons are shown. Data are represented as means ± S.E.M; *p < 0.05, **p < 0.01, ***p < 0.001).

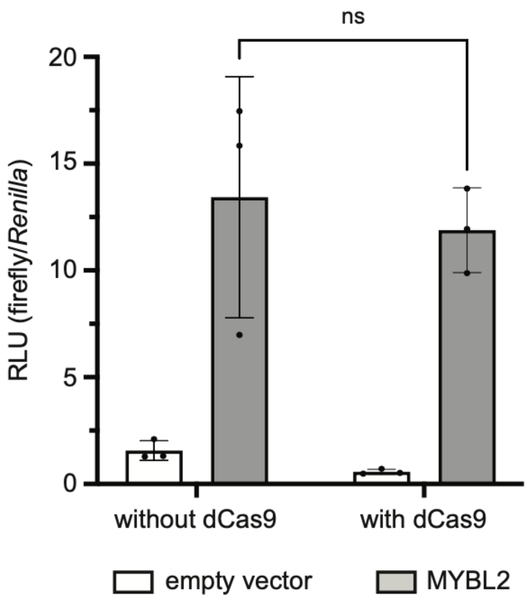

Supplementary Figure S4: **Lack of modulation of *ADGB* promoter-driven luciferase activity by dCas9 in absence of transiently transfected gRNAs.** Luciferase reporter gene assay in Hek293T cells transfected with empty pGL3b vector or MYBL2 with or without dCas9 in the

absence of gRNA. Transiently transfected dCas9 alone, without a gRNA does not decrease *ADGB* promoter-driven luciferase activity. Data are represented as means ± S.E.M.

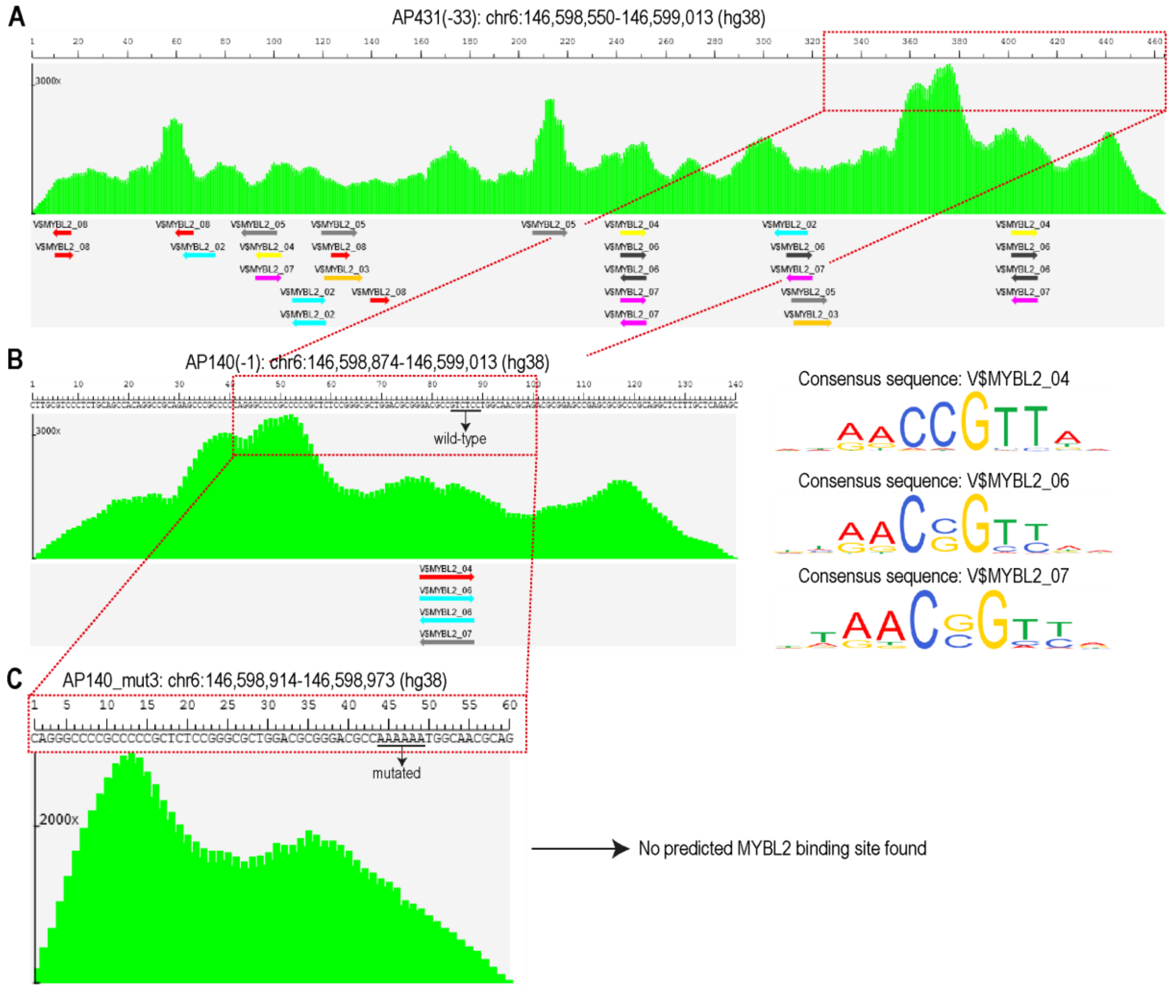

D

AP140(-1)

ID	Sequence	From	To	Length	Strand	Type	Property: coreScore	Property: score	Property: siteModel
...	...	...	...	...	...	...	...	...	...
6650	AP140(-1)	16	27	12	+	TF binding site	0.82187	0.7766	V$MYCMAX_02
6635	AP140(-1)	17	26	10	+	TF binding site	0.79398	0.82638	V$MXI1_05
6636	AP140(-1)	46	59	14	+	TF binding site	0.78654	0.61139	V$MYBL1ELF1_01
6639	AP140(-1)	65	77	13	-	TF binding site	0.72646	0.76005	V$MYBL1EOMES_02
6640	AP140(-1)	70	91	22	+	TF binding site	0.62179	0.66786	V$MYBL1MAX_02
6641	AP140(-1)	75	91	17	+	TF binding site	0.89056	0.69316	V$MYBL1_03
6649	AP140(-1)	75	91	17	+	TF binding site	0.91575	0.72263	V$MYB_05
6642	AP140(-1)	78	88	11	+	TF binding site	0.92663	0.80751	V$MYBL1_07
6644	AP140(-1)	78	89	12	-	TF binding site	0.6	0.67397	V$MYBL1_09
6645	AP140(-1)	78	88	11	+	TF binding site	0.83403	0.71793	V$MYBL2_04
6646	AP140(-1)	78	88	11	+	TF binding site	0.9014	0.78108	V$MYBL2_06
6647	AP140(-1)	78	88	11	-	TF binding site	0.8866	0.77097	V$MYBL2_06
6648	AP140(-1)	78	88	11	-	TF binding site	0.90082	0.75404	V$MYBL2_07
6643	AP140(-1)	91	102	12	+	TF binding site	0.8	0.68404	V$MYBL1_09
6638	AP140(-1)	93	106	14	-	TF binding site	0.82271	0.60877	V$MYBL1ELF1_01
6637	AP140(-1)	101	114	14	+	TF binding site	0.57307	0.64304	V$MYBL1ELF1_01
...	...	...	...	...	...	...	...	...	...

E

AP140_mut3

ID	Sequence	From	To	Length	Strand	Type	Property: coreScore	Property: score	Property: siteModel
...	...	...	...	...	...	...	...	...	...
2829	AP140_mut3	6	19	14	+	TF binding site	0.78654	0.61139	V$MYBL1ELF1_01
2830	AP140_mut3	25	37	13	-	TF binding site	0.72646	0.76005	V$MYBL1EOMES_02
2833	AP140_mut3	26	45	20	+	TF binding site	0.6	0.66756	V$MYCMAX_03
2828	AP140_mut3	28	40	13	-	TF binding site	0.80714	0.69205	V$MXI1_04
2831	AP140_mut3	37	53	17	-	TF binding site	0.60575	0.67265	V$MYBL1_08
2832	AP140_mut3	45	54	10	+	TF binding site	0.78074	0.80649	V$MYB_07
...	...	...	...	...	...	...	...	...	...

No predicted MYBL2 binding site found

Supplementary Figure S5: **Predicted MYBL2 binding sites in the *ADGB* promoter are lost after mutating AP140 sequence.** Predicted binding sites for MYBL2 transcription factor, within **(A)** AP431(-33) [AP431], (**B)** AP140(-1) [AP431_C] and **(C)** AP140_mut3 [AP431_C**mut3**] sequences, are reported based on TRANSFAC predictions. The MYBL2 consensus binding sequences predicted within AP140(-1) are represented based on TRANSFAC database. A summary table for predicted binding sites for MYBL2 within AP140(-1) sequence is also displayed **(D)**. No binding sites for MYBL2 were predicted within AP140_mut3 **(E)**.

A

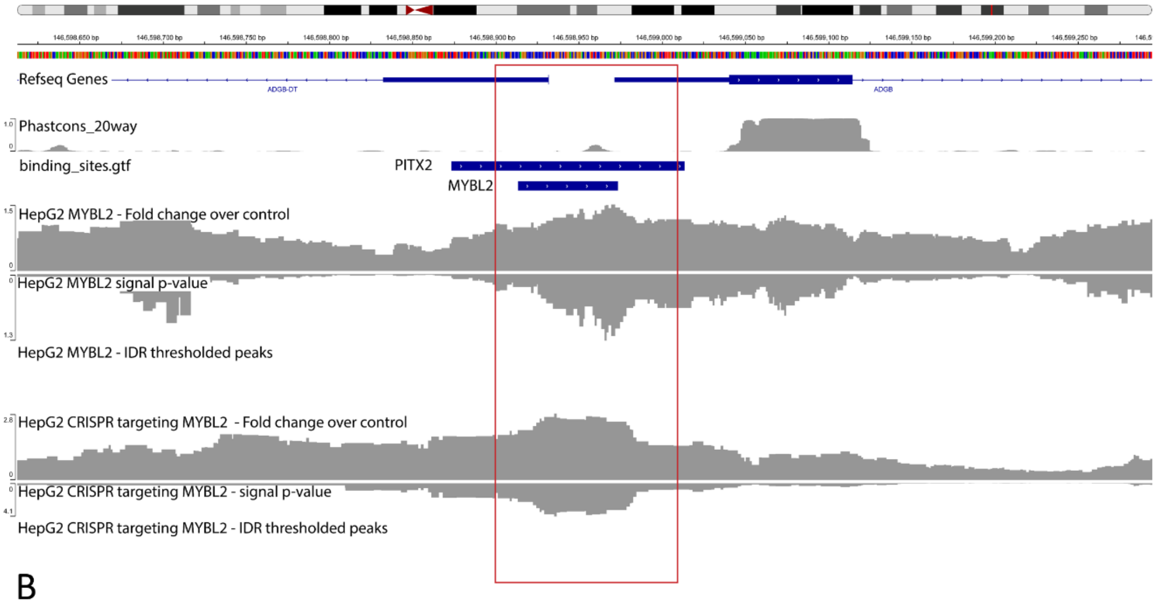

B

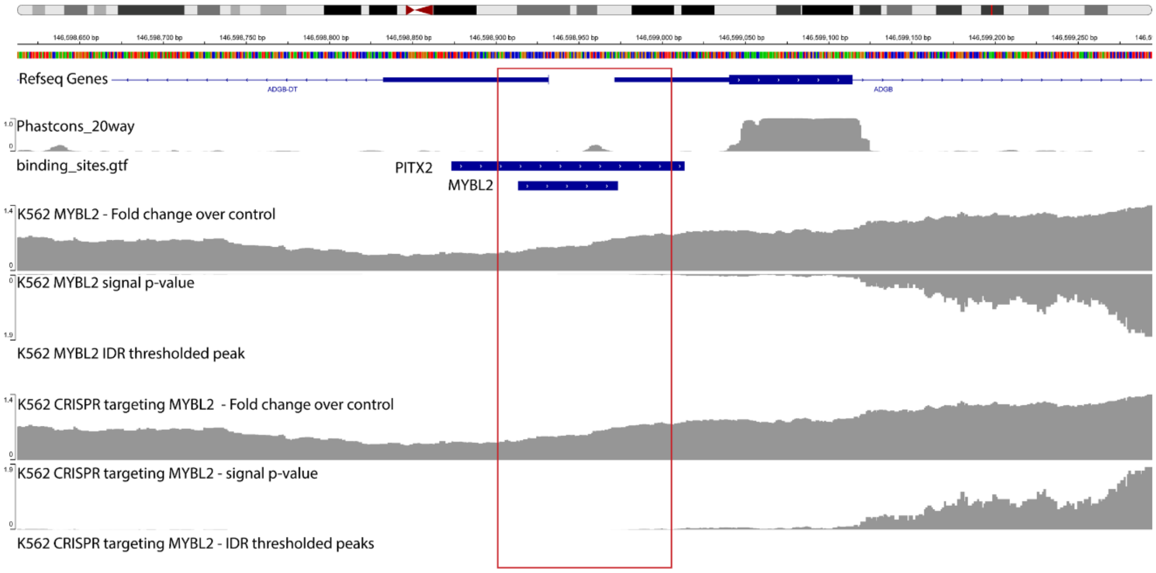

Supplementary Figure S6: **Analysis of publicly available ENCODE ChIP-sequencing data**. Data was visualized with the IGV browser. Depicted are the fold change of sequence coverage compared to the IG input, the corresponding p-value and high confidence peaks. The range containing the putative binding sites for MYBL2 and PITX2 are represented as blue bars in the highlighted area. Both binding areas encompass a conserved region. A) Sequencing data of a MYBL2 pulldown in wildtype HepG2 cells and cells with a CRISPR-targeted *MYBL2* locus. There is no significant peak called in the area over the *ADGB* promoter, however, there is an increase in fold change over control that encompasses the experimentally validated binding site of MYBL2. The signal is not abolished in the CRISPR targeted cell line. This enrichment of sequences seems to be cell line dependent since a similar experimental setup in K562 cells (B) shows no enrichment of sequences at this locus.

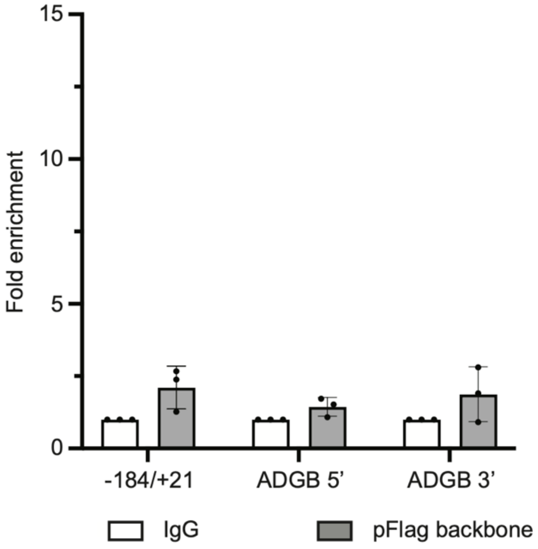

Supplementary Figure S7: **Chromatin immunoprecipitation in non-transfected cells displays absence of MYBL2 or PITX2 binding at the *ADGB* locus**. Chromatin immunoprecipitation (ChIP) experiment in HEK293T cells transiently transfected with the empty pFLAG vector. Coprecipitated chromatin derived from the *ADGB* promoter was determined by qPCR using a primer pair covering +21 bp to – 184 bp upstream of the *ADGB* TSS. Control regions were targeted upstream (5' end, ADGB 5') and downstream (3'end, ADGB 3') of *ADGB* on chromosome 6. Data are represented as means ± S.E.M.

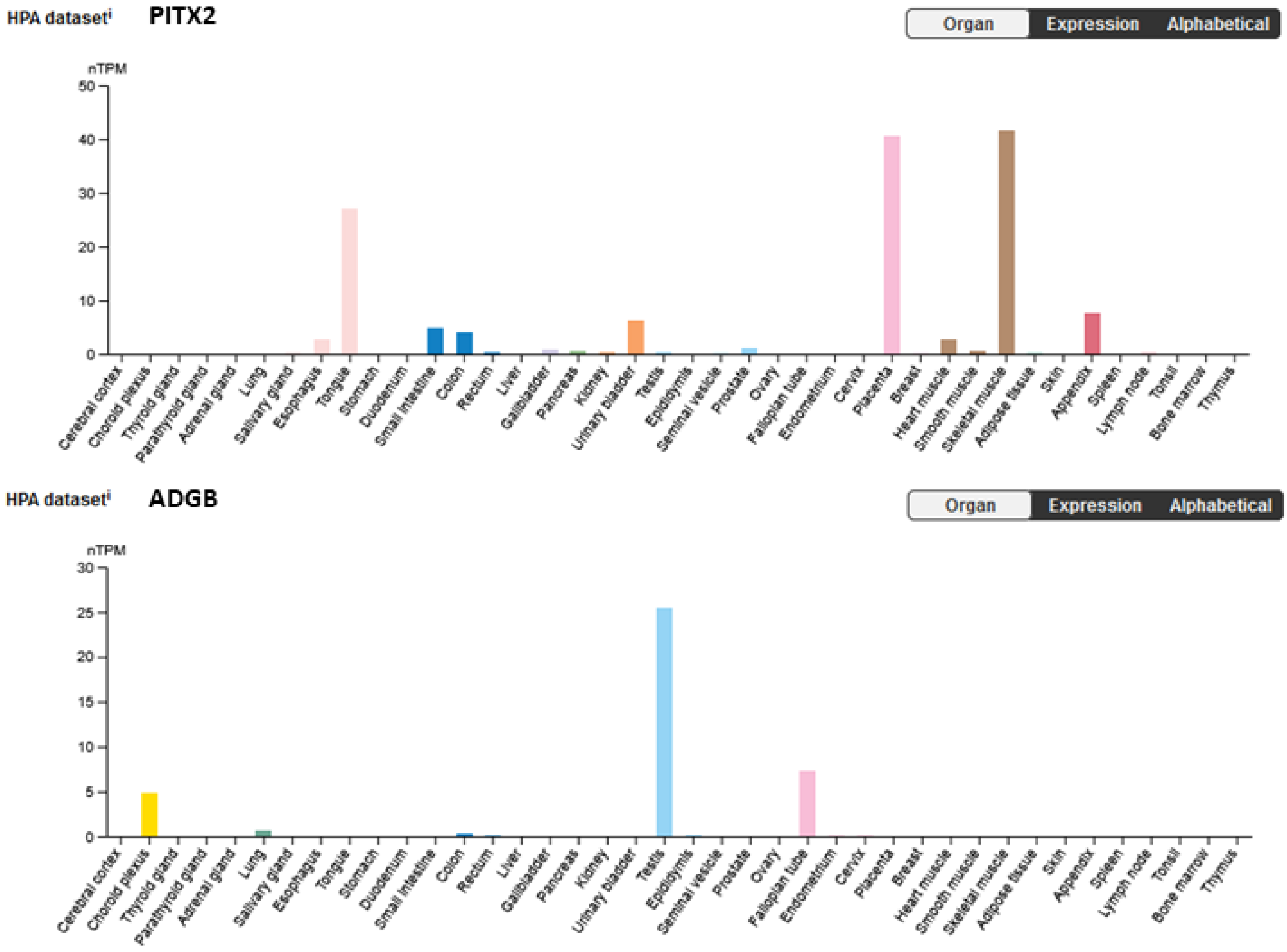

Supplemental Figure S8: **mRNA expression of PITX2 and ADGB derived from the Human Protein Atlas.** PITX2 and ADGB mRNA do not co-occur in any of the tissues analysed.

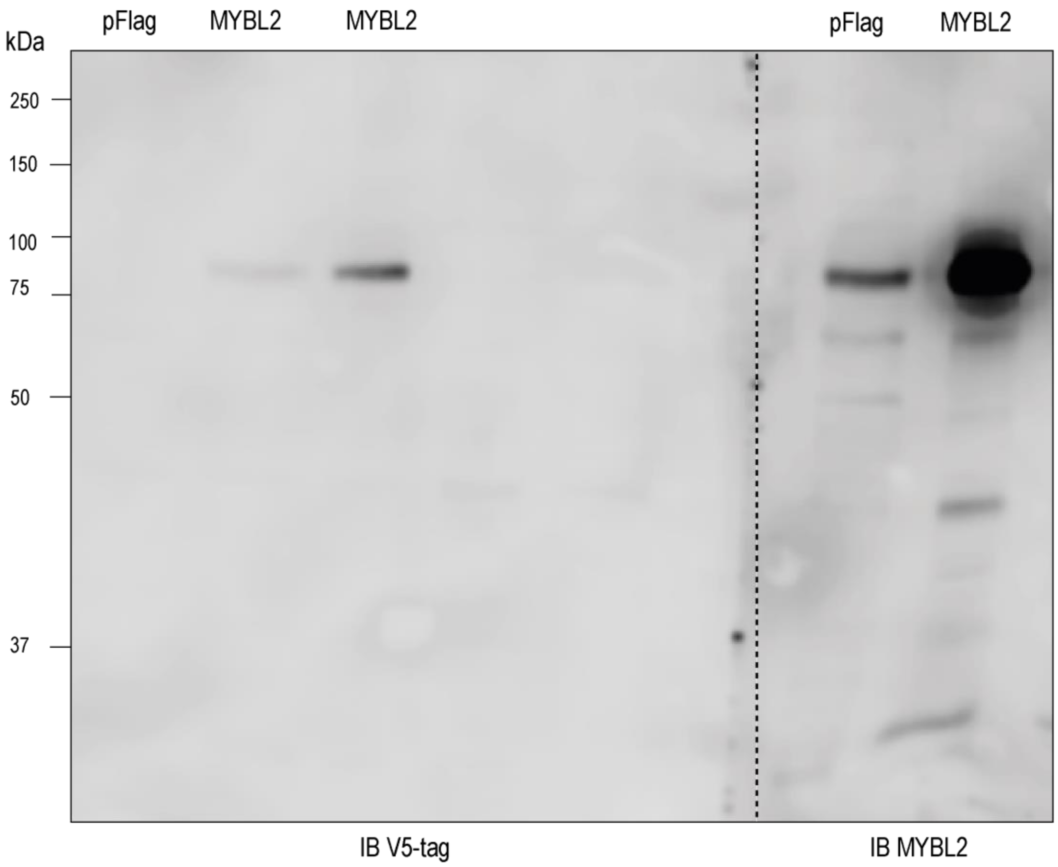

Supplementary figure S9: **Endogenous MYBL2 expression in HEK293T.** Endogenous MYBL2 protein levels were verified by immunoblotting with MYBL2 and V5-tag specific antibodies. pLenti6.2/V5-DEST-MYBL2 or an empty pFLAG vector were overexpressed in HEK293T cells. Molecular size of the V5-tag signal (left) corresponds to high intensity bands in blots treated with anti-MYBL2 antibody. A strong overexpression of MYBL2 can be observed, but also a clear band resulting from endogenous expression in the sample transfected with the empty pFLAG vector.
